# Association between sarcopenia and atrial fibrillation: a meta-analysis

**DOI:** 10.3389/fmed.2026.1853470

**Published:** 2026-07-29

**Authors:** Peng Wang, Wentao Shi, Zhenze Yu, Guojie Ye, Yingyue Zhang, Chunhua Ding

**Affiliations:** 1 Cardiac Department, Aerospace Center Hospital, Peking University Aerospace School of Clinical Medicine, Beijing, China; 2 Department of Cardiology, The Seventh People's Hospital of Zhengzhou, Zhengzhou, China

**Keywords:** arrhythmia, atrial fibrillation, meta-analysis, sarcopenia, system evaluation

## Abstract

**Background:**

Sarcopenia, a condition characterized by age-related muscle mass loss, has been recognized as closely associated with the onset of various cardiovascular diseases. Although some studies suggest that sarcopenia may be associated with an increased risk of atrial fibrillation (AF), the evidence supporting this association remains insufficient. This study aims to systematically evaluate existing clinical research to explore the relationship between sarcopenia and atrial fibrillation.

**Methods:**

A systematic search was conducted of relevant literature in the PubMed, Embase, Cochrane Library, and Web of Science databases. The search period ranged from the establishment of the databases to May 15, 2025. Inclusion criteria were observational studies that explored the relationship between sarcopenia and atrial fibrillation. All included studies will be assessed for quality using the Newcastle-Ottawa Scale (NOS) to evaluate the quality of study selection, comparison, and results, ensuring the reliability of the analysis results. The data for this study will be analyzed using Stata 15.

**Results:**

A total of 6 studies involving 3,067,241 patients, Meta-analysis results suggest that sarcopenia is associated with a higher likelihood of developing AF compared to patients without sarcopenia [OR = 2.16, 95% CI (1.80, 2.60)]. Multivariate regression results suggest that sarcopenia is associated with a higher likelihood of developing AF [OR = 1.93, 95% CI (1.40, 2.66)].

**Conclusion:**

The study suggests a significant association between sarcopenia and AF, indicating that sarcopenia may be linked to an increased risk of AF. However, given the observed heterogeneity and potential publication bias, more high-quality research is needed to further substantiate these findings and confirm the nature of this relationship.

## Background

Atrial fibrillation (AF) is a common sustained arrhythmia, and its prevalence continues to rise globally (1, 2). According to the latest report from the American Heart Association (AHA), the prevalence of AF in the elderly population aged 60 and above can reach over 10%, with a significant increase as age advances (3). AF not only significantly increases the risk of stroke but is also closely associated with adverse clinical outcomes such as heart failure, cognitive impairment, and mortality (4). Risk factors for AF include hypertension, diabetes, coronary artery disease, heart failure, and lifestyle factors such as smoking (5). However, recent study suggests that age-related factors, such as sarcopenia, may play a crucial role in the development and progression of AF (6).

Sarcopenia is a chronic disease caused by multiple factors, characterized by a decline in skeletal muscle mass and strength (7). As people age, muscle mass gradually decreases, and this condition is most observed in individuals over the age of 60 (8). According to the definition by the European Working Group on Sarcopenia in Older People (EWGSOP), sarcopenia not only refers to a reduction in muscle mass but also includes a decrease in muscle strength and impaired physical function (9). The diagnosis of sarcopenia typically relies on a comprehensive assessment of muscle mass, muscle strength, and functional capacity. The development of sarcopenia is closely associated with factors such as aging, chronic diseases, malnutrition, and reduced physical activity (10). Studies indicate that the prevalence of sarcopenia in individuals aged 65 and older ranges from approximately 5–13% and can exceed 30% in those aged 70 and older (11, 12). Sarcopenia has been proven to be an independent factor contributing to reduced quality of life, increased risk of falls and fractures in the elderly, and is also closely associated with cardiovascular diseases, metabolic disorders, and diabetes (13).

Increasing evidence suggests that sarcopenia may be associated with an elevated risk of AF, but this hypothesis has not yet been systematically validated (14, 15). Therefore, exploring the relationship between sarcopenia and AF and elucidating its underlying mechanisms is of great significance for understanding the pathogenesis of AF and providing new insights for clinical intervention. In recent years, there has been an increasing number of studies investigating the association between sarcopenia and AF. Several observational studies and cohort studies have reported that patients with sarcopenia have a higher risk of developing AF (16). A comprehensive analysis of these studies revealed that the impact of sarcopenia on AF varies across different populations. For example, the risk increase is particularly pronounced in men and older age groups, and some studies suggest that sarcopenia plays a predictive role in the development of AF. Additionally, some studies indicate that the association between sarcopenia and AF is time-dependent, with prolonged muscle loss potentially further increasing the risk of AF (17, 18).

Currently, research on the relationship between sarcopenia and AF lacks systematic analysis. Most studies focus on single variables or small populations and have not yet explored the underlying mechanisms in different populations in depth. Therefore, this study not only contributes to the growing body of evidence suggesting an association between sarcopenia and AF but also provides new insights that may inform future clinical prevention and treatment strategies, pending further validation of this relationship. By exploring the relationship between sarcopenia and AF, we can provide theoretical basis for clinicians in the early screening, risk assessment, and development of intervention strategies for AF.

## Methods

The study accordance with the Preferred Reporting Items for Systematic Reviews and Meta-Analyses (PRISMA) guidelines (19). This study was pre-registered with the International prospective register of systematic reviews (PROSPERO) under the registration number: CRD420251054023.

### Inclusion criteria

The inclusion criteria: study participants must be adults aged 18 years or older, and participants must have a confirmed diagnosis of sarcopenia (according to the criteria of the European Sarcopenia Working Group or the American Geriatrics Society), with comparisons made to a control group without sarcopenia. The study types include cross-sectional studies, cohort studies. The diagnostic criteria for arrhythmia must be clearly defined, with the type of arrhythmia confirmed using methods such as electrocardiography (ECG) or Holter monitoring. The study must provide sufficient statistical data, odds ratios (OR), or relative risks (RR) along with their 95% confidence intervals. Additionally, the included literature must be publicly published and peer-reviewed studies, with no language restrictions for this study.

### Exclusion criteria

Exclusion criteria include studies without a control group, those with methodological flaws, those lacking sufficient data, or those that are merely case reports or review articles. Additionally, studies focusing on intervention effects (pharmacological or physical therapy) rather than natural associations will be excluded. These criteria ensure that the included studies have high quality and reliability, facilitating a systematic assessment of the relationship between sarcopenia and arrhythmia through subsequent meta-analysis.

### Literature retrieval

A comprehensive literature search was conducted in PubMed, Embase, Web of Science, and the Cochrane Library from database inception to May 15, 2025.

The search strategy combined Medical Subject Headings (MeSH) and free-text terms related to “sarcopenia” and “atrial fibrillation.” The PubMed search strategy was as follows: (“sarcopenia” [MeSH Terms] OR sarcopenia [Title/Abstract] OR muscle loss [Title/Abstract]) AND (“atrial fibrillation” [MeSH Terms] OR atrial fibrillation [Title/Abstract] OR AF [Title/Abstract]). Equivalent search strategies were adapted for Embase, Web of Science, and the Cochrane Library using appropriate database-specific syntax. No language restrictions were applied. The detailed search strategy for each database is provided in Supplementary Table S1.

### Study collection and extraction

Two authors must independently screen the retrieved studies, excluding duplicates and those that do not meet inclusion criteria by reading titles and abstracts. They will review the full text of each study to select those that qualify. Any disagreements will be resolved through discussion with a third reviewer. Data extraction from the included studies will be carried out independently by two reviewers using a predefined data acquisition list. This list will capture basic information (author, year and country of publication), study design, sample size, gender (male/female), mean age, diagnosis of sarcopenia, and regression model. If necessary, a third reviewer will double-check the data for consistency. If data is missing or incomplete, we will contact the study authors to obtain it. Studies without available data will be excluded.

### Assessment of risk of bias

The risk of bias in the included studies was evaluated independently by two investigators, and the results will be cross-checked. For cohort and case–control studies, the Newcastle Ottawa Scale [NOS (20)] was used to assess quality. The NOS evaluates studies based on three dimensions: population selection, comparability, and exposure or outcome, with eight items totaling nine points. Scores range from 0 to 4 (low quality), 5–6 (moderate quality), and 7–9 (high quality). Studies scoring 0–4 was excluded. For cross-sectional studies, this study was used the AHRQ quality (21) assessment tool to evaluate quality. This tool primarily assesses the rationality of the study design, the representativeness of the sample selection, the clarity of the definitions of exposure and outcome, the accuracy of the data collection process, the rationality of the statistical methods, and the completeness of the report.

### Statistical analysis

Stata 15.0 was used for statistical analysis, sensitivity evaluation, and publication bias assessment. Pooled odds ratios (ORs) with 95% confidence intervals (CIs) will be calculated using a random-effects model to assess the association between sarcopenia and atrial fibrillation, as reported in the included studies. Heterogeneity was assessed using Cochrane’s *Q* test and the *I*^2^ statistic. A *p*-value > 0.05 for *Q* and *I*^2^ < 50% will indicate statistical homogeneity, with random-effects models employed for all meta-analyses. The between-study variance (*τ*^2^) will be estimated using the Der Simonian and Laird method, with the restricted maximum likelihood (REML) method as an alternative. The quality of evidence was assessed using the GRADE approach, where observational studies begin with moderate certainty, downgraded for bias or inconsistency but upgraded with large effect sizes. Publication bias was evaluated using funnel plot asymmetry, Egger’s regression test, and Harbord’s test. If small-study effects or publication bias are detected, the trim-and-fill method was applied to adjust for missing studies. Sensitivity analyses were assessed the robustness of results, including excluding small studies or those with extreme effect sizes. Meta-regression was performed as an exploratory and hypothesis-generating analysis to investigate potential sources of heterogeneity, including publication year, country, sample size, mean age, and regression model.

## Results

### Literature search results

**Figure 1:**
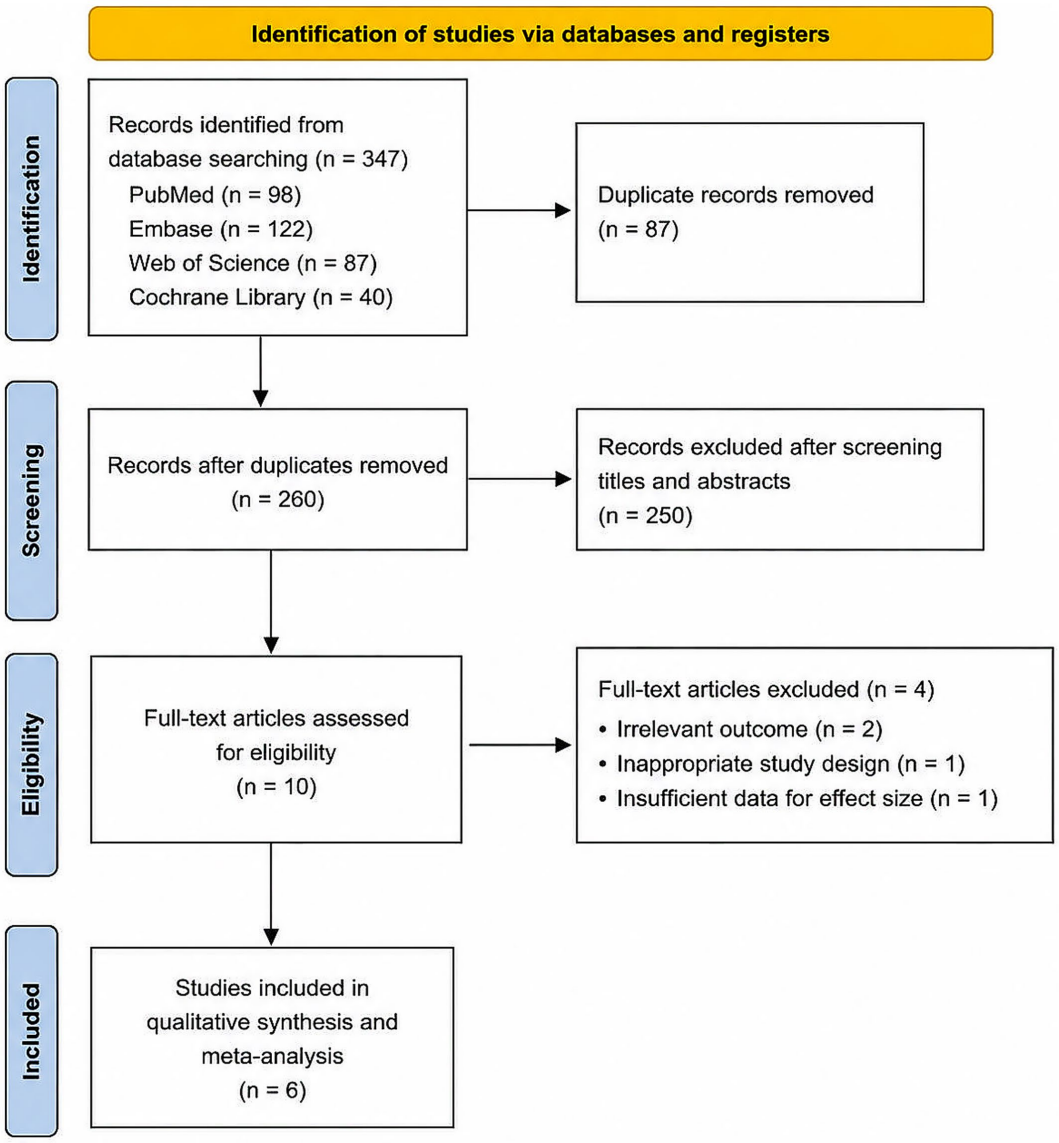
Literature search flow chart.

A total of 347 records were identified through database searching. After removing 87 duplicate records, 260 records were screened by title and abstract. Of these, 250 records were excluded. A total of 10 full-text articles were assessed for eligibility, and 4 studies were excluded for reasons such as irrelevant outcomes or study design. Finally, 6 observational studies (22–27) were included in the meta-analysis (Figure 1).

### Basic characteristics of included studies

This study included a total of 6 studies involving 3,067,241 patients, including 4 cohort studies (22, 23, 25, 26) and 2 cross-sectional studies (24, 27), with ages ranging from 48.58 to 76.5 years. The definition of sarcopenia varied across included studies, including muscle mass-based criteria (e.g., ASM/height^2^), muscle strength-based assessment (grip strength), physical performance measures, predicted body composition indices, and composite scoring systems. AF was ascertained using different methods across included studies, including electrocardiography (ECG), Holter monitoring, medical records, and International Classification of Diseases (ICD) codes from administrative databases. These variations were systematically extracted and summarized in Table 1.

**Table 1:** Basic characteristics of the included studies.

Study	Year	Study design	Country	Sample size	Gender (M/F)	Mean age	Diagnosis of sarcopenia	AF ascertainment	Regression model
Kılıç	2024	Cohort study	Turkey	722	306/416	70.1	Sarcopenia score of ≥105 in men and ≥120 in women	ICD-10 claims + medical records	Logistic regression
Yu	2025	Cohort study	China	4,321	2,302/2,019	68.05	SMM/BW (<38.2% for mem and <32.2% for women)	UK Biobank: ICD codes + hospital records + death registry	Logistic regression
Shim	2024	Cross-sectional	Korea	2,225	1,018/1,207	76.5	ASM/height2 < 7.0 kg/m^2^ for men and <5.4 kg/m2 for women	Claims database (ICD-based)	Logistic regression
Tang	2024	Cohort study	China	384,433	175,510/208,923	58	ASM/height2 < 7.0 kg/m^2^ for men and <5.4 kg/m^2^ for women	ECG + clinical diagnosis	Logistic regression
Woo	2023	Cohort study	Kore	2,673,108	1,375,197/1,287,929	48.58	ASM/height2 < 7.0 kg/m^2^ for men and <5.4 kg/m^2^ for women	ECG-based diagnosis	Cox regression
Xia	2021	Cross-sectional	China	2,432	992/1,440	62.2	ASM/height2 < 7.0 kg/m^2^ for men and <5.4 kg/m^2^ for women	ECG + medical history	Logistic regression

### Risk of bias

The methodological quality of the included studies is summarized in Table 2. For cohort studies, the Newcastle–Ottawa Scale (NOS) item-level assessment showed generally high methodological quality, with scores ranging from 8 to 9 stars. Most studies performed well in participant selection and outcome assessment domains; however, some limitations were observed in the comparability domain due to incomplete adjustment for potential confounders. For cross-sectional studies, the overall quality was rated as moderate according to the AHRQ assessment tool. The main limitations were related to selection bias and limited control of confounding variables. According to the GRADE approach (Supplementary Table S2), the overall certainty of evidence for the association between sarcopenia and atrial fibrillation was rated as low. This was primarily due to the observational nature of included studies, substantial heterogeneity, and potential publication bias. Therefore, the results should be interpreted with caution.

**Table 2:** Risk of bias results.

Cross-sectional											
Study	Whether the source of the information is clear	Whether exposed and non-exposed groups are listed	Whether a time was given to identify patients	If not, population derived, whether the subjects were consecutive	Whether the subjective factors of the evaluator cover up other aspects of the research object	Any assessment performed to ensure quality is described	The rationale for excluding any patients from the analysis was explained	Describe measures to evaluate and/or control for confounding factors	Explain how missing data were handled in the analysis	Response rates and the completeness of data collection are summarized	If there is follow-up, identify the percentage of patients with expected incomplete data or follow-up results
Shim et al. (23)	Yes	Unclear	Yes	Yes	Yes	Yes	Yes	Yes	Yes	Unclear	Yes
Tang et al. (24)	Yes	Unclear	Unclear	Unclear	Yes	Yes	Yes	Yes	Yes	Unclear	Yes

Cohort study									
Study	Representativeness of the exposed group	Selection of non-exposed groups	Determination of exposure factors	Identification of outcome indicators not yet to be observed at study entry	Comparability of exposed and unexposed groups considered in design and statistical analysis	design and statistical analysis	Adequacy of the study’s evaluation of the outcome	Adequacy of follow-up in exposed and unexposed groups	Total scores
Kılıç et al. (22)	*	*	*	*	**	*	*	*	9
Yu et al. (27)	*	*	*	*	**	*	*	*	9
Shim et al. (23)	*	*	*	/	**	*	*	*	8
Tang et al. (24)	*	*	*	/	**	*	*	*	8

*one score, **two scores.

### Meta-analysis

#### Occurrence of atrial fibrillation in sarcopenia

**Figure 2:**
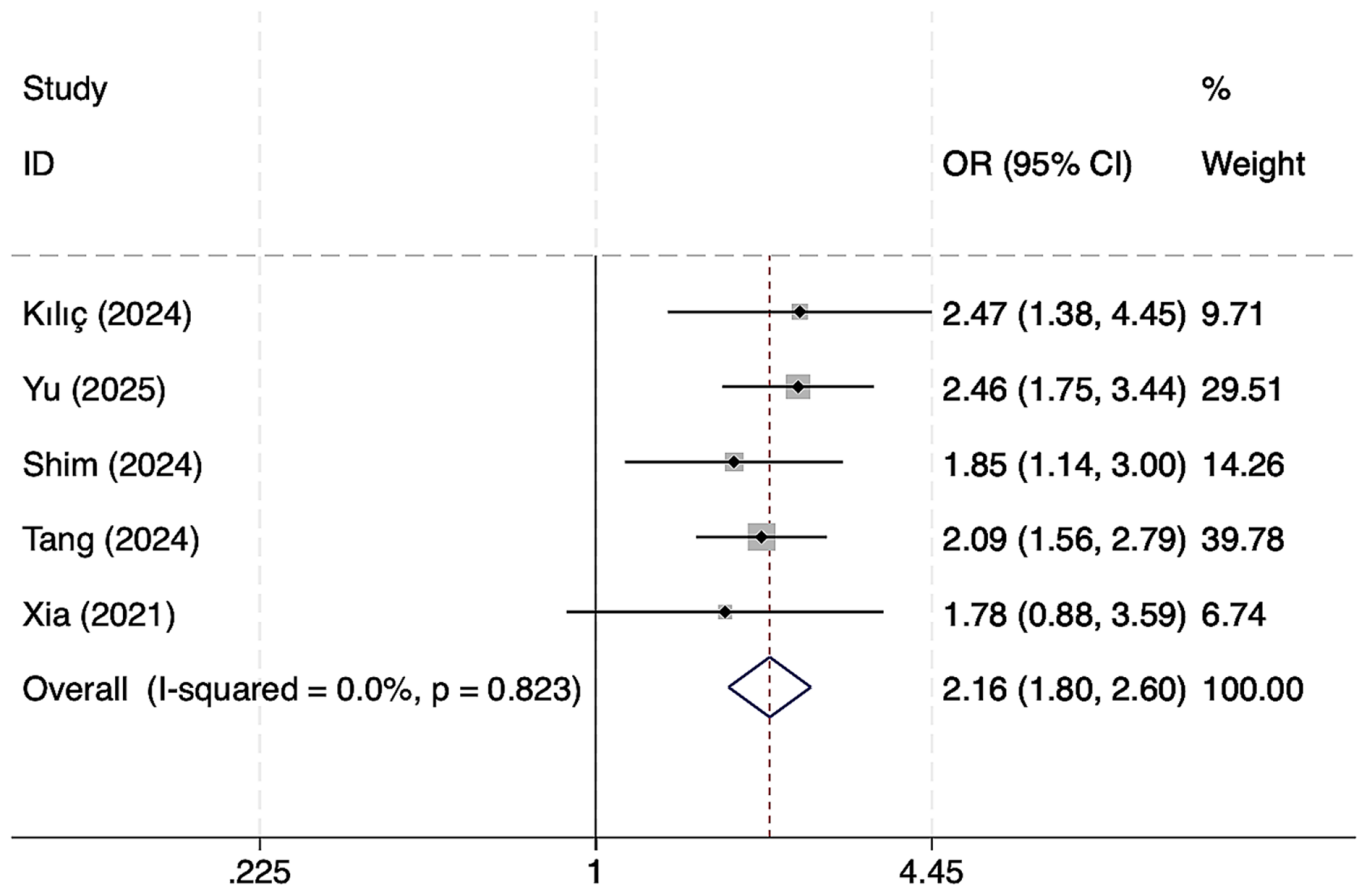
Meta-analysis forest plot of occurrence of atrial fibrillation in sarcopenia.

Five articles in this study mentioned the incidence of AF in patients with sarcopenia. Heterogeneity testing (*I*^2^ = 0%, *p* = 0.823) was performed using a fixed-effect model. The results (Figure 2) suggest that patients with sarcopenia were more likely to develop AF than patients without sarcopenia [OR = 2.16, 95% CI (1.80, 2.60)].

#### Association of sarcopenia with atrial fibrillation

**Figure 3:**
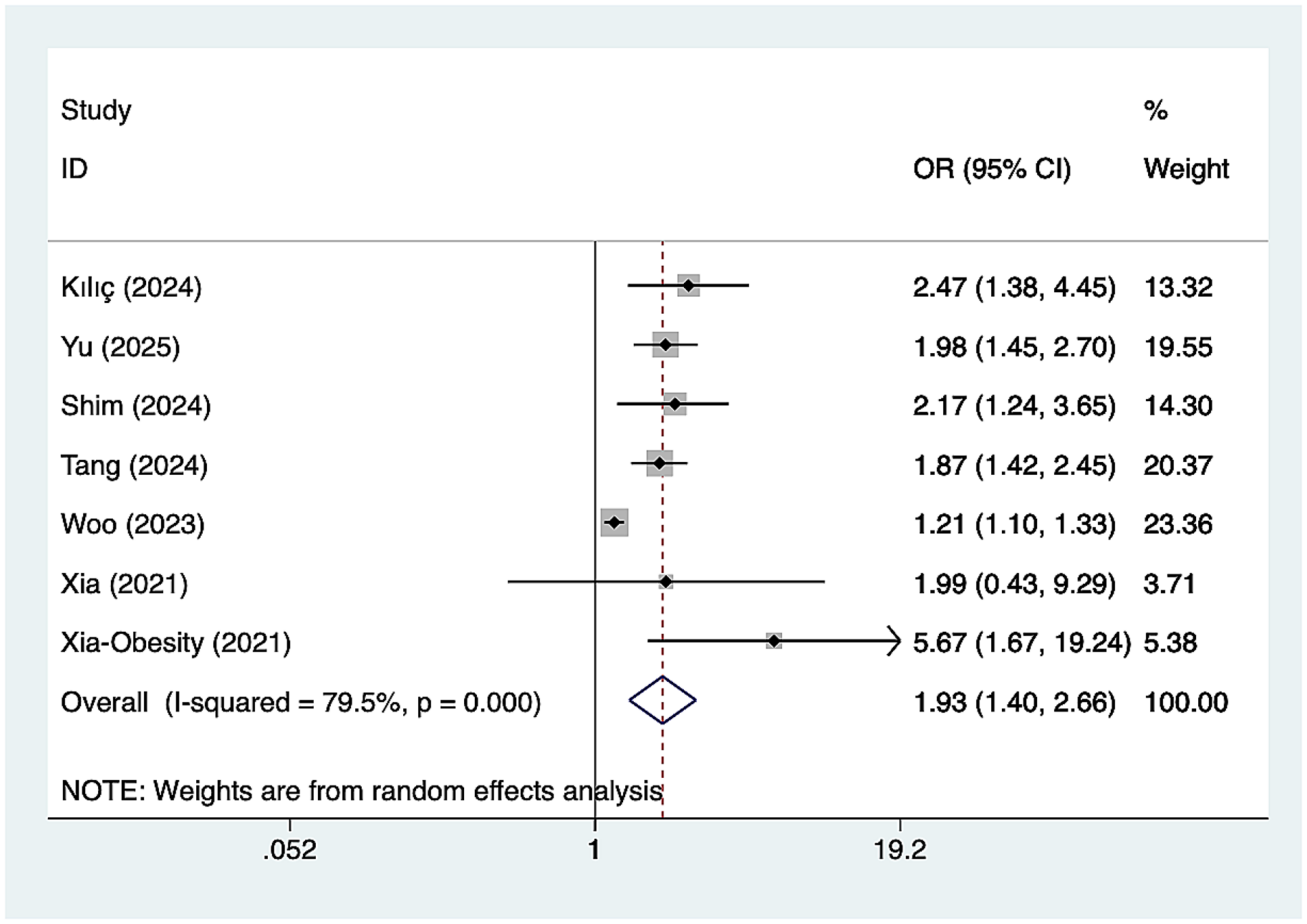
Forest plot of meta-analysis of association of sarcopenia with atrial fibrillation.

**Figure 4:**
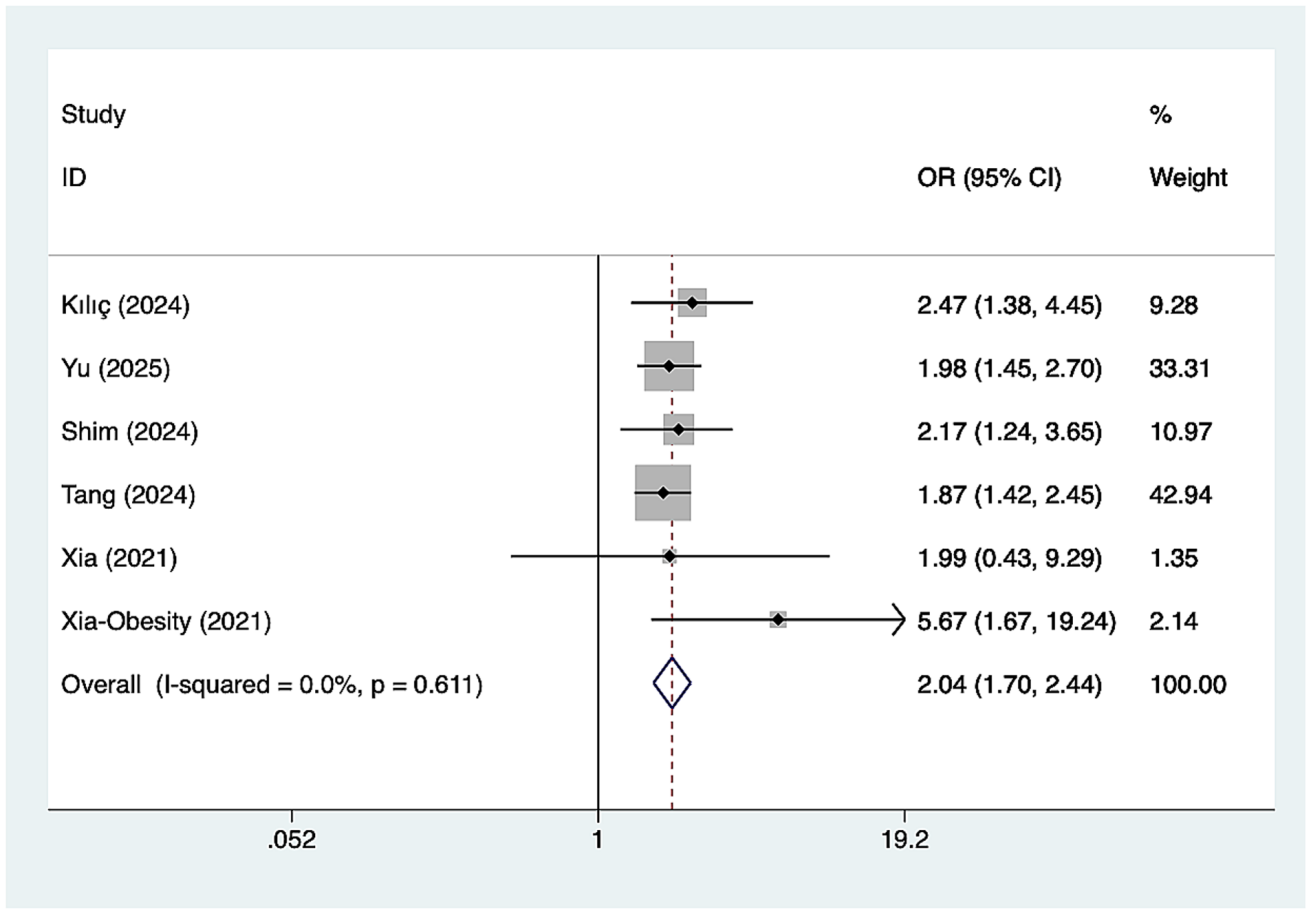
Forest plot of meta-analysis of association of sarcopenia with atrial fibrillation excluding the Woo 2023 study.

This study collected multivariate regression analysis results on sarcopenia and AF from six studies. Among them, XIA2021 was divided into obese and non-obese groups, thus forming two separate studies. Heterogeneity testing (*I*^2^ = 79.5%, *p* = 0.001) was conducted using a random-effects model. The analysis results (Figure 3) indicate that patients with sarcopenia were associated with AF [OR = 1.93, 95% CI (1.40, 2.66)]. Due to significant heterogeneity, sensitivity analysis was conducted by sequentially excluding studies. The results (Supplementary Figure S1) suggest that heterogeneity may originate from the Woo 2023 study. In sensitivity analyses, exclusion of the Woo 2023 study (Figure 4) reduced heterogeneity; however, the direction of association remained consistent, and the overall conclusion was not materially altered [OR = 2.04, 95% CI (1.70, 2.44)].

#### Meta regression

Meta-regression was conducted as an exploratory analysis due to the limited number of included studies (*n* = 6). The analysis results (Table 3) suggested potential contributions of publication year, country, sample size, mean age, and regression model to heterogeneity; however, these findings should be interpreted with caution due to low statistical power.

**Table 3:** Meta-regression results.

Variable	Coef	Std. err	*p*	95% CI
Year of publication	0.485	0.156	0.023	[0.085, 0.885]
Study design	0.564	0.865	0.544	[−1.661, 2.788]
Country	0.700	0.238	0.032	[0.089, 1.312]
Sample size	0.291	0.232	0.019	[0.434, 1.626]
Mean age	0.045	0.014	0.021	[0.101, 0.807]
Diagnosis of sarcopenia	0.340	0.631	0.613	[−1.282, 1.963]
Regression model	0.757	0.223	0.019	[0.183, 1.332]

#### Subgroup analysis results

Subgroup analyses (Supplementary Table S3) were conducted according to study design to address methodological heterogeneity between cohort and cross-sectional studies.

For the outcome of occurrence of atrial fibrillation, cohort studies consistently demonstrated a significant association between sarcopenia and atrial fibrillation (pooled effect = 1.86, 95% CI: 1.72–2.01), with relatively low to moderate heterogeneity. In contrast, cross-sectional studies also showed a significant association (pooled effect = 2.93, 95% CI: 1.72–5.00), although with higher heterogeneity across studies. For the outcome of overall association between sarcopenia and atrial fibrillation, similar patterns were observed. Cohort studies showed a stable association (pooled effect = 1.91, 95% CI: 1.78–2.05), while cross-sectional studies demonstrated a stronger but more variable association (pooled effect = 2.61, 95% CI: 1.45–4.70).

#### Publication bias

Funnel plots and Egger’s tests were used to explore potential publication bias. For the outcome of “occurrence of atrial fibrillation in sarcopenia,” the funnel plot (Supplementary Figure S2) appeared relatively symmetrical, and Egger’s test was not statistically significant (*p* = 0.675). For the outcome of “association between sarcopenia and atrial fibrillation,” asymmetry was observed in the funnel plot (Supplementary Figure S3), and Egger’s test suggested potential small-study effects (*p* = 0.009). A trim-and-fill sensitivity analysis (Supplementary Figure S4) was performed; however, these results were considered exploratory given the limited number of included studies.

## Discussion

This study, combining data from six observational studies, indicates a significant association between sarcopenia and an increased risk of AF, suggesting that sarcopenia may be a risk marker or associated condition for AF. These findings provide important guidance for clinical practice, particularly in the elderly population, where early identification of sarcopenia could help inform strategies for preventing or managing AF.

### Key findings

In this study, data from six observational studies indicated that patients with sarcopenia had a significantly increased risk of developing AF. Specifically, the odds ratio for AF in patients with sarcopenia was 2.16, indicating that the risk of AF was more than twice as high in patients with sarcopenia compared to those without sarcopenia. Further analysis of the results of multivariate regression analysis also showed similar findings, with the presence of sarcopenia in patients significantly increasing the risk of AF, with an OR of 1.93. These findings are consistent with existing literature (15) and provide strong support for sarcopenia associated with AF. However, although the overall analysis indicates a significant association between sarcopenia and AF, the results exhibit high heterogeneity (*I*^2^ = 79.5%,), which may be attributed to differences in study design and sample characteristics across studies. The presence of heterogeneity suggests that caution is warranted when interpreting the results, and they should not be simply regarded as universally applicable conclusions. Therefore, this study further conducted a sensitivity analysis to exclude the study with high heterogeneity (Woo 2023 study). After excluding this study, the heterogeneity significantly decreased (*I*^2^ = 0%), and the effect size of the results was strengthened (OR = 2.04). This change suggests that the design and sample characteristics of specific studies may have a significant impact on overall results, so it is important to consider all factors comprehensively when conducting systematic evaluations.

### Heterogeneity analysis

The heterogeneity analysis in this study revealed significant differences among the studies, particularly in terms of study design, sample sources. Heterogeneity is a common issue in meta-analyses, especially when involving observational studies. We further explored the sources of heterogeneity through meta-regression analysis. The results indicated that publication year, study country, sample size, mean age, and different choices of regression models may be the primary factors contributing to heterogeneity. Future studies should strive to adopt uniform standards for these factors to minimize the influence of confounding variables. Sample size and mean age may directly impact the stability and generalizability of study results. For example, smaller sample sizes may lead to greater variability in statistical outcomes, while larger sample sizes typically provide more robust conclusions. Additionally, the age range of the study population may influence the interpretation of results, particularly in older adults, where sarcopenia prevalence is higher, and physiological characteristics and underlying conditions in older adults may exacerbate the association between sarcopenia and AF (28). Therefore, future studies should implement more rigorous control of these variables to further validate the authenticity of sarcopenia associated with AF. The Woo 2023 study differed from other included studies in several aspects, including its extremely large sample size, different population source (nationwide database), and use of hazard ratio based on Cox regression. These differences may partly explain its contribution to heterogeneity. However, the primary conclusions were based on the full dataset, and sensitivity analyses were performed only to assess robustness.

### Publication bias

Publication bias is a significant issue in systematic reviews that can affect the generalizability of study results. This study used funnel plots and Egger’s test to detect publication bias and found no significant publication bias in the association between sarcopenia and AF incidence. However, publication bias was observed in the association between sarcopenia and AF. To further explore this issue, this study employed trim-and-fill methods for bias correction, and the results suggested that even if publication bias exists, the corrected conclusions remain reliable. The presence of publication bias suggests that some relevant studies may not have been published or included in the analysis, and these omitted studies may influence the overall results. Particularly in observational studies related to cardiovascular diseases such as AF, reporting bias may exist. Therefore, researchers should strive to minimize such biases when designing and writing studies to ensure the comprehensiveness and accuracy of the results.

### Biological mechanisms

The potential biological mechanisms linking sarcopenia and AF may involve multiple factors. Sarcopenia patients often exhibit systemic inflammatory responses, increased oxidative stress, and metabolic disorders (29). These factors may contribute to the development of AF by affecting cardiac electrophysiology. For example, sarcopenia is often associated with chronic low-grade inflammation (30). Studies have shown that inflammatory markers such as C-reactive protein (CRP) and interleukin-6 (IL-6) are significantly elevated in patients with AF (31). These markers are believed to potentially lead to changes in atrial structure and ultimately contribute to the development of AF. Sarcopenia itself may also cause changes in the neuromuscular system, thereby affecting the balance of autonomic nervous system function (32). Changes in autonomic nervous system function may lead to increased sympathetic nervous activity and reduced parasympathetic nervous activity, which have important implications for cardiac electrophysiological stability and are one of the key factors contributing to the onset of AF (33).

### Clinical significance

This study suggests a significant association between sarcopenia and AF, so clinical screening and intervention for sarcopenia should be strengthened, especially in elderly patients. Clinicians can identify patients with sarcopenia early and implement appropriate exercise interventions and nutritional management to help reduce the incidence of AF. This strategy is of great importance for the prevention and management of cardiovascular diseases, particularly in the elderly population, where the prevention and treatment of sarcopenia may yield significant health benefits. Interventions targeting sarcopenia, such as muscle-building exercises, moderate aerobic exercise, and nutritional supplementation (protein, vitamin D, and calcium), have been proven to effectively improve muscle mass and function in patients. Exercise interventions aimed at enhancing muscle strength can improve patients’ physical performance, slow the progression of sarcopenia, and thereby potentially reduce the risk of AF. Therefore, clinicians should encourage patients to engage in targeted exercise and nutritional interventions, especially among the elderly population, to reduce the incidence of AF.

### Strengths and limitations

This systematic review and meta-analysis has several strengths. First, the study addresses a clinically relevant question regarding the association between sarcopenia and atrial fibrillation, which is of importance in geriatrics, cardiology, and preventive medicine. Second, a relatively large pooled sample size was achieved by including all available observational studies to date. Third, the study followed PRISMA guidelines and was prospectively registered in PROSPERO, enhancing transparency and methodological rigor. Fourth, a comprehensive search strategy was applied using multiple major databases. Finally, we systematically assessed study quality and performed subgroup, sensitivity, and publication bias analyses to explore potential sources of heterogeneity and evaluate the robustness of the findings.

Several limitations of this meta-analysis should be acknowledged. First, all included studies were observational in design, which limits the ability to infer causality. Consequently, reverse causation cannot be excluded, as atrial fibrillation may also contribute to the development or progression of sarcopenia. In addition, residual confounding remains a concern because unmeasured or incompletely adjusted variables may have influenced the observed associations.

Second, there was substantial methodological heterogeneity across the included studies. This included differences in study design (cohort and cross-sectional studies), as well as variability in the definitions and diagnostic criteria of sarcopenia. Sarcopenia was assessed using different approaches, including muscle mass-based indices, muscle strength measures, physical performance tests, predicted body composition models, and composite diagnostic criteria. Such variability may introduce classification bias and affect the comparability and generalizability of the findings.

Third, the methods used to ascertain atrial fibrillation also varied across studies, including electrocardiography, medical record review, and administrative claims data. These differences in outcome ascertainment may have introduced detection bias and contributed to heterogeneity.

Fourth, the relatively small number of included studies limited the statistical power of subgroup analyses, meta-regression, and publication bias assessments. Therefore, these exploratory analyses should be interpreted with caution.

Finally, publication bias may be present, as only published studies were included in the analysis, and studies with null or negative findings may be underrepresented. Overall, although these limitations may influence the magnitude of the pooled estimates, they are unlikely to alter the overall direction of the observed association between sarcopenia and atrial fibrillation.

## Conclusion

This systematic review and meta-analysis demonstrated that sarcopenia is significantly associated with an increased risk of atrial fibrillation. However, due to the observational nature of the included studies, a causal relationship cannot be established. These findings should be interpreted as an association rather than evidence of causation. Further large-scale prospective studies are needed to confirm these results.

## Data Availability

The original contributions presented in the study are included in the article/Supplementary material, further inquiries can be directed to the corresponding author.
