# Supplementary material for: Association between sarcopenia and atrial fibrillation: a meta-analysis
Source: Front Med (Lausanne). 2026 Jul 29;13:1853470. doi: 10.3389/fmed.2026.1853470 (PMC13461522; doi:10.3389/fmed.2026.1853470)
Supplement: Supplementary file 1 [file Data_Sheet_1.DOCX]

Table S1 specific search strategy

|  | search strategy |
| --- | --- |
| PubMed | (("Sarcopenia"[MeSH Terms] OR sarcopenia[Title/Abstract] OR "muscle loss"[Title/Abstract] OR "skeletal muscle loss"[Title/Abstract] OR "low muscle mass"[Title/Abstract] OR "muscle wasting"[Title/Abstract])  AND  ("Atrial Fibrillation"[MeSH Terms] OR "atrial fibrillation"[Title/Abstract] OR AF[Title/Abstract] OR "atrial flutter"[Title/Abstract])) |
| Embase | ('sarcopenia'/exp OR sarcopenia:ti,ab OR 'muscle loss':ti,ab OR 'skeletal muscle atrophy':ti,ab OR 'low muscle mass':ti,ab)  AND  ('atrial fibrillation'/exp OR 'atrial fibrillation':ti,ab OR af:ti,ab OR 'atrial flutter':ti,ab) |
| Cochrane library | (sarcopenia OR "muscle loss" OR "low muscle mass" OR "skeletal muscle atrophy")  AND  ("atrial fibrillation" OR AF OR "atrial flutter") |
| Web of science | TS=(sarcopenia OR "muscle loss" OR "skeletal muscle loss" OR "low muscle mass" OR "muscle wasting")  AND  TS=("atrial fibrillation" OR AF OR "atrial flutter") |

Table S2 GRADE Summary of Findings

| Outcome | Studies | Effect | Certainty (GRADE) | Reasons for downgrade |
| --- | --- | --- | --- | --- |
| AF occurrence | 5 studies | OR 2.16 (1.80–2.60) | Low | Observational design, heterogeneity |
| AF occurrence | 5 studies | OR 2.16 (1.80–2.60) | Low | Observational design, heterogeneity |

Table S3 Subgroup analysis of sarcopenia and atrial fibrillation

| Subgroup | Outcome | No. of studies | Model | Pooled effect | 95% CI | Heterogeneity | Interpretation |
| --- | --- | --- | --- | --- | --- | --- | --- |
| Cohort studies | Occurrence of AF | 4 | Random effects | 1.86 | 1.72–2.01 | Low–moderate | Significant positive association |
| Cross-sectional studies | Occurrence of AF | 3 | Random effects | 2.93 | 1.72–5.00 | High | Strong but heterogeneous association |
| Cohort studies | Association of AF | 4 | Random effects | 1.91 | 1.78–2.05 | Low | Stable positive association |
| Cross-sectional studies | Association of AF | 3 | Random effects | 2.61 | 1.45–4.70 | High | Strong but variable association |

Figure S1 Sensitivity analysis of Association of sarcopenia with atrial fibrillation.

Figure S2 Funnel plot of Occurrence of atrial fibrillation in sarcopenia

Figure S3 Funnel plot of the Association of sarcopenia with atrial fibrillation

Figure S4 trim-and-fill results for the association of sarcopenia with atrial fibrillation
